# Temporal Dynamics of Influenza A(H5N1) Subtype before and after the Emergence of H5N8

**DOI:** 10.3390/v13081565

**Published:** 2021-08-07

**Authors:** Fatma Amer, Ruiyun Li, Neveen Rabie, Mohamed H. El-Husseiny, Nahed Yehia, Naglaa M. Hagag, Mohamed Samy, Abdullah Selim, Mohamed K. Hassan, Wafaa M. M. Hassan, Abdel-Sattar Arafa, Åke Lundkvist, Momtaz A. Shahein, Mahmoud M. Naguib

**Affiliations:** 1 Reference Laboratory for Veterinary Quality Control on Poultry Production, Animal Health Research Institute, Agriculture Research Center, 12618 Giza, Egypt; fatmaamer29@gmail.com (F.A.); nevo_talk@yahoo.com (N.R.); olivera_2006@yahoo.com (M.H.E.-H.); nahedyehia@gmail.com (N.Y.); naglaahagagahri@gmail.com (N.M.H.); mohamedsamy_@hotmail.com (M.S.); AbdullahSelim@yahoo.com (A.S.); mkahassan2020@gmail.com (M.K.H.); fooaaa@love.com (W.M.M.H.); araby85@hotmail.com (A.-S.A.); momtaz.shahein@yahoo.com (M.A.S.); 2 Centre for Ecological and Evolutionary Synthesis (CEES), Department of Biosciences, University of Oslo, N-0316 Oslo, Norway; ruiyun.li@ibv.uio.no; 3 Zoonosis Science Center, Department of Medical Biochemistry and Microbiology, Uppsala University, 75121 Uppsala, Sweden; ake.lundkvist@imbim.uu.se

**Keywords:** avian influenza virus, H5N1, H5N8, co-circulation, Egypt

## Abstract

Highly pathogenic avian influenza (HPAI) viruses continue to circulate worldwide, causing numerous outbreaks among bird species and severe public health concerns. H5N1 and H5N8 are the two most fundamental HPAI subtypes detected in birds in the last two decades. The two viruses may compete with each other while sharing the same host population and, thus, suppress the spread of one of the viruses. In this study, we performed a statistical analysis to investigate the temporal correlation of the HPAI H5N1 and HPAI H5N8 subtypes using globally reported data in 2015–2020. This was joined with an in-depth analysis using data generated via our national surveillance program in Egypt. A total of 6412 outbreaks were reported worldwide during this period, with 39% (2529) as H5N1 and 61% (3883) as H5N8. In Egypt, 65% of positive cases were found in backyards, while only 12% were found in farms and 23% in live bird markets. Overall, our findings depict a trade-off between the number of positive H5N1 and H5N8 samples around early 2017, which is suggestive of the potential replacement between the two subtypes. Further research is still required to elucidate the underpinning mechanisms of this competitive dynamic. This, in turn, will implicate the design of effective strategies for disease control.

## 1. Introduction

Avian influenza virus (AIV) continues to cause outbreaks in different bird species and pose a public health concern [1]. At least four influenza pandemics have occurred in the last 100 years, which have emerged from the avian virus gene pool [2,3]. Outbreaks of the highly pathogenic avian influenza (HPAI) virus among poultry populations have resulted in devastating economic damages to the poultry industry worldwide [4]. In addition, HPAI viruses have the ability to cross the species barrier and infect humans and other mammals, posing a potential pandemic threat [5]. The documented HPAI viruses, H5N1 and H5N8, are fundamental subtypes, dominating the outbreaks among bird species in several countries over the last two decades.

In 1996, HPAI H5N1 virus was first detected in a goose farm in southern China [6]. Since then, the virus has spread globally and formed diverse transmission sources and pathways [7,8]. The virus continued to evolve via mutations and/or reassortment with other AIV subtypes, and diverged into 10 distinct phylogenetic clades (clade 0–9) and several subcaldes [9]. The HPAI H5N1 virus has shown the ability to infect humans and was firstly reported in humans in 1997 in Hong Kong [10]. The virus has been reported to cause a total of 862 cases, including 455 deaths [11]. In 2005, a considerable H5N1 outbreak was reported in migratory birds at Qinghai Lake, China [12]. Subsequently, closely related viruses (of clade 2.2) were transmitted to other countries across Asia, Europe, the Middle East, and Africa [13]. This has been associated with a negative impact on the local poultry industry, including, for example, economic losses as well as losses of meat and egg sources. Currently, HPAI H5N1 virus is still endemic in several countries, including China, Egypt, Vietnam, Indonesia, India, and Bangladesh [14,15,16,17], and causes periodical outbreaks in several other countries in Asia, Europe, and Africa [18,19,20].

In parallel, HPAI H5N8 virus was reported in several countries in Asia and spread as far as to Europe and North America, infecting wild birds, domestic poultry, and zoo birds during its first wave in 2014 (clade 2.3.4.4.) [21,22]. In 2016/2017, another novel reassortant of HPAI H5N8 virus was reported in Russia. Different genotypes of this virus (carrying variable internal gene segments) were subsequently identified and disseminated via the migration of wild birds to many countries in Europe, Asia, and Africa within a few months, causing the most widespread epidemic by HPAI virus since the H5N1 epidemic in 2005 [23,24]. In 2020–2021, HPAI H5N8 viruses were again spreading rapidly, and were responsible for >1000 outbreaks in 25 countries, mainly in Europe [25,26]. The new HPAI H5N8 virus was reported to have genetic similarity and phylogenetic relatedness with H5N8 viruses currently circulating in Egypt [25]. Moreover, HPAI H5N8 was reported in humans in February 2021 (seven poultry farm workers, in Russia) with a previous history of contacting infected birds [11]; however, no symptoms, as well as no evidence of onward human-to-human transmission, was found.

Recent studies have shown co-circulation and co-infection of both HPAI H5N1 and H5N8 subtypes in Europe and the Middle East [16,25]. However, studying the temporal dynamics of HPAI H5N1 virus circulation before and after the emergence of HPAI H5N8 virus is very limited. Here, we study the temporal dynamics of the co-circulation of two HPAI subtypes—H5N1 and H5N8—on a global scale and in Egypt, which is a clear example of the co-circulation of the two subtypes. In Egypt, HPAIV H5N1 has been endemic since 2006, causing hundreds of outbreaks among different poultry species, and was responsible for 359 human infections (with 120 fatalities) [11,27]. In late 2016, HPAI H5N8 virus was first reported in Egypt [28] and, since then, both subtypes have been co-circulating [29,30]. Therefore, using data collected from Egypt in 2014–2020, we investigated the correlation of H5N1 dynamics before and after the emergence of H5N8.

## 2. Materials and Methods

### 2.1. Data Source and Spatial Analysis

We established the global H5N1 and H5N8 dataset by retrieving data of reported outbreaks in January 2015 to October 2020 from the Global Animal Disease Information System (Source: FAO. EMPRES-i. H5N1 and H8N8 flu dataset. URL: https://empres-i.review.fao.org/#/ (accessed on 27 April 2021). Reproduced with permission). The retrieved dataset includes outbreaks reported to WHO, OIE, and Promed, as well as cases retrieved from publications. For each outbreak, we collected the spatio-temporal information (i.e., spatial location and observed date) and the virus subtype. To identify statistically significant spatial clusters of H5N1 and H5N8, we used the Getis-Ord Gi* statistic to examine the spatial cluster of both large (or hot spots) and small (or cold spots) numbers of the documented outbreaks. We further classified the hot and cold spots to differing levels of significance at the 99, 95, and 90 percent confidence level. The spatial statistics are implemented in ArcGIS 10.3.

Additionally, we established a dataset for the investigation of the spatio-temporal distribution of H5N1 and H5N8 subtypes in Egypt. Since 2006, systemic surveillance has been implemented under the supervision of the reference laboratory for quality control on poultry production (RLQP), the Animal Health Research institute, and the General Organization for Veterinary Services in Egypt. This surveillance covers multiple sectors, including commercial farms, backyards sectors, and live bird markets (LBMs). Epidemiological data available at RLQP was retrieved from January 2014 to October 2020, where a total number of 76,966 samples collected from farms (63,412, 82.4%), backyards (11,281, 14.8%), and LBMs (2134, 2.8%) were tested for influenza A virus. To characterize the dynamics of the spatial distribution of the two subtypes, we aggregated the annual number of positive H5N1 and H5N8 samples in each governorate and visualized their spatio-temporal distribution in ArcGIS 10.3. Additionally, we aggregated the positive samples for each subtype on a finer, i.e., monthly, scale in Egypt. These monthly data were used for the correlation analysis below.

### 2.2. Correlation Analysis

To investigate the temporal correlation of H5N1 and H5N8, we made a rolling correlation analysis of the number of positive samples of the two subtypes. Assuming a window size of 9, we initialized the analysis by estimating the Pearson’s product moment correlation coefficient, such that the correlation at one month is estimated using the number of positive samples from four months before to four months after. Given that the correlation coefficient is highly dependent on the size of the rolling window, we further validated the findings by varying the window size from 3 to 45. For each window size, we projected the correlation coefficients over time. Across the sizes, we plotted the distribution of the timing for maximum correlation estimates. We implemented the analysis on varying spatial scales. First, we investigated the overall temporal correlation of the two subtypes using the number of outbreaks worldwide. Further, we examined the variation of correlation in different species, i.e., domestic vs. wild birds. Next, we examined the temporal correlation using positive samples from all sectors in Egypt. To examine the variation of the correlation across various sectors, we estimated the correlation coefficient in commercial farms, backyards sectors, and LBMs separately. The statistical analysis was made in stats package in R (v3.3.3) [31].

## 3. Results

### 3.1. Surveillance Outcomes

**Figure 1:**
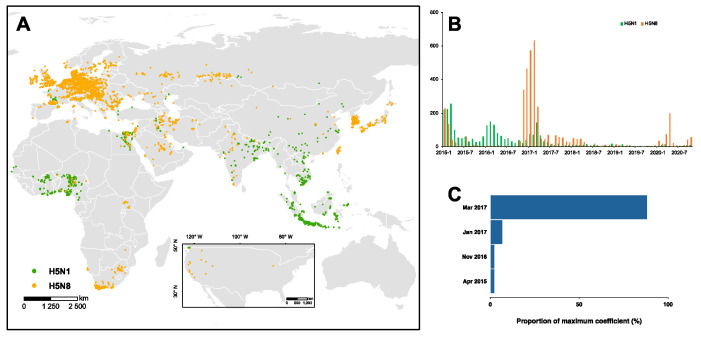
H5N1 and H5N8 outbreaks in 2015–2020. (**A**) Spatial and (**B**) temporal distribution of the two subtypes on the globe. (**C**) The timing of the maximum correlation of the dynamics of two subtypes is identified by correlation analysis. Across the vast range of the window size for the correlation analysis, the distribution of the timing for maximum coefficient is presented.

Globally, a total of 6412 outbreaks were found, with 39% (2529) designated as H5N1 and the remaining 61% (3883) as H5N8 (Figure 1A). Our findings show a clear spatial clustering of both subtypes (Figures S1 and S2). Throughout 2015–2020, 60% of the H5N1 outbreaks were documented in Africa (1514 out of 2529), with hot spots located in Egypt and West Africa (Figure S1), while approximately 70% of the H5N8 outbreaks were reported in Europe (2678 out of 3883) together with multiple spatial clusterings of high and low number of outbreaks (Figure S2). In Egypt, our surveillance reported a total of 1.6% (1256 out of 76,966) tested samples as H5N1/N8 positive among the 26 governorates. Of the positive samples, 74% (934) were H5N1 and 26% (322) were H5N8. The majority (65%) of the positive samples were isolated from backyards, while only 12% and 23% positive samples were isolated from farms and LBMs, respectively. Additionally, the number of positive samples decreased over time, with approximately 74% of the positive cases obtained in the initial three years (i.e., 2014–2016). Across the governorates, Dakahlia, Giza, and Minya isolated over 100 positive samples, which collectively consisted of 25% of the total positive samples. Furthermore, we observed a strong seasonality in 2014, with a typically larger number of positive samples during December to February, while, during other years, we isolated a small number of positive samples and a relatively mild seasonality.

### 3.2. Changing Temporal and Spatial Distribution of H5N1 and H5N8

Throughout 2015–2020, there have been three waves of HPAI H5N1/N8 viruses with distinct temporal distribution (Figure 1B). The first wave, driven by H5N1, was documented in 2015, diminishing over the following years. Of note, we demonstrated that such diminishing is highly associated with the big outbreak of H5N8 in 2016–2017, and successively since 2020 (Figure 1C). Similarly, the transition of dominance from H5N1 to H5N8 in domestic birds may have occurred around November 2016. However, such transition is insignificant in wild birds due, primarily, to the limited number of outbreaks (Figure S3). Across the regions, we identified that this association of the two subtypes is typical among African countries; the majority of the H5N1/N8 outbreaks (47%) are reported in Egypt. Therefore, we focused on Egypt to investigate the changing spatio-temporal distribution of H5N1 and H5N8 using our long-term, large-scale surveillance data.

**Figure 2:**
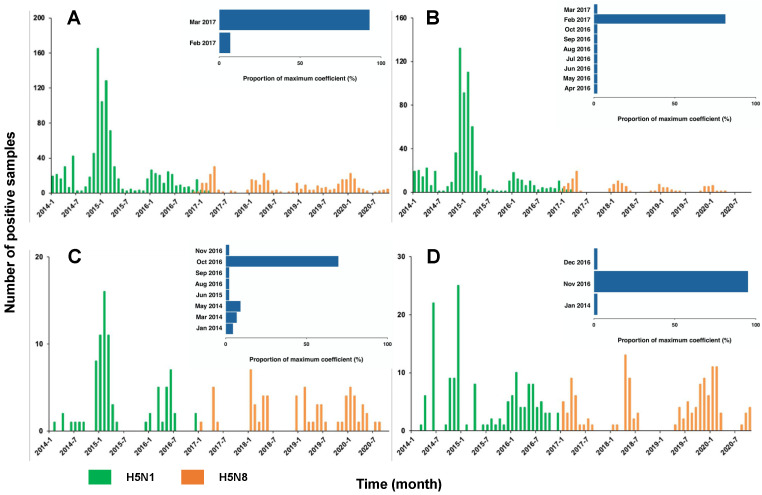
Numbers of positive HPAI H5N1 vs. HAPI H5N8 cases in Egypt 2014–2020. (**A**) Overall, (**B**) backyards, (**C**) farms, and (**D**) LBMs. The insets show the distribution of the timing for the maximum correlation of two subtypes.

**Figure 3:**
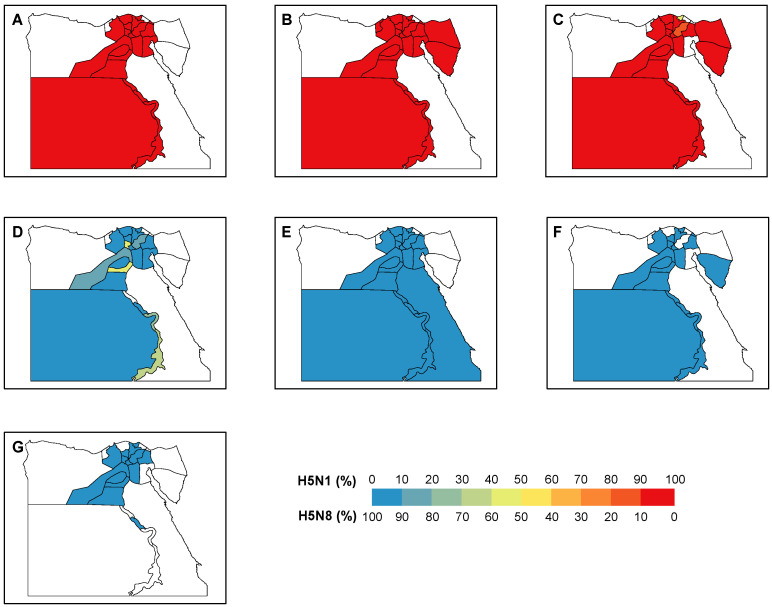
Distribution of H5N1 and H5N8 positive samples in Egypt. The proportions of positive sample of each subtype are shown by governorate in each year during (**A**–**G**) 2014–2020.

Overall, there was a trade-off between the number of positive H5N1 and H5N8 samples around the end of 2016/early 2017 (Figure 2A). During the period 2014–2016, 100% of the positive samples were H5N1, which revealed the dominance of H5N1 before the emergence of H5N8. Since 2017, this dominance was replaced by H5N8 with only 2% (7 out of 320) H5N1 positive samples. With the majority (40 out of 43) of window sizes used in the analyses, the maximum correlation coefficients were expected in March 2017. This is suggestive of March 2017 as the critical timing for the transition of the dominance between two subtypes. We showed a similar trade-off between the two subtypes across the various locations, i.e., the backyards, farms, and LBMs (Figure 2B–D). This trade-off was validated by the correlation analyses, indicating the highest correlation of the dynamics of the two subtypes around late 2016/early 2017 (Figure 2B–D, Figure S4). Consistent with the changing distribution over time, our findings demonstrated a clear transition of the dominance over the governorates (Figure 3). Across the governorates, H5N8 accounted for >90% of the positive samples in Egypt during the period 2014–2016 (Figure 3A–C). Of note, the number of positive H5N1 samples greatly decreased, accounting for a maximum of 50% among all governorates in 2017 (Figure 3D). This is in line with the emergence of H5N8, which constituted at least 50% of all the positive samples. H5N8 has become the primary subtype of HPAI in Egypt since 2017 (Figure 3E–G).

## 4. Discussion

Avian influenza viruses of subtypes H5N1 and H5N8 have represented the vast majority of reported HPAI subtypes in birds during the last two decades [32], posing a continuous threat to the poultry industry. Several countries have reported co-circulation of both subtypes in different bird species [25]. Sharing the same host population can lead to competition and impact on the survival of one or both of the virus types [33]. Understanding the implications that one subtype of AIV might have on the circulation of another subtype has become a top priority to improve our ability to fight AIV. Egypt is located at the crossroads of the East Africa–West Asia and the Mediterranean/Black Sea migratory flyways facing Europe and Asia. This important location highlights Egypt as a hotspot for virus introduction and spread from Eurasia to Africa and vice versa [34]. Recently, the new 2020/2021 HPAI H5N8 variant revealed a genetic similarity and phylogenetic relatedness with the H5N8 viruses currently circulating in Egypt [25]. Different genotypes of HPAI H5N8 virus have been reported in Egypt since its first isolation in late 2016 [35,36,37]. Moreover, Egypt is one of the countries with the highest poultry density, which may explicate the endemicity of AIV. Given this, we performed our analyses globally and feature Egypt with the aim to highlight the transition of the dominance of the two fundamental AIV subtypes in shaping viral dynamics on multiple spatial scales.

Our findings indicate that March 2017 is the critical period in the transition of dominance between H5N1 and H5N8 across countries, Egyptian governorates, and different sectors. The potential impact of HPAI H5N8 on the circulation of HPAI H5N1 detected in this study raises critical questions about the driving factors responsible for this replacement. Did the HPAI H5N1 virus acquire one or more mutations that impacted its transmissibility/virulence? Do HPAI H5N8 viruses of clade 2.3.4.4b have the ability to compete with HPAI H5N1 inside the host cell? Animal experimental studies, in both the natural host (mallard) and domestic poultry (chicken), are required to understand the impact of H5N8 on H5N1. Relatedly, the mechanism underlying the interactions of two subtypes is the key direction for future studies. With evidence of the transition of dominance between two subtypes, stochastic modelling may be the tool to use to include the underlying mechanisms and driving factors to capture how the shift in the dominant subtypes shape the overall epidemic dynamics [38].

Moving beyond the transition of dominance, our findings shed a key light on the big H5N8 outbreaks in the most recent months. It is worth noting that there have been around 1500 H5N8 outbreaks in Europe (75% of all global H5N1 and H5N8 outbreaks) since November 2020. This results in a strong economic burden through its huge impact on the poultry industry and culling of millions of birds. Both subtypes have been reported to transmit from wild aquatic birds to domestic poultry and vice versa [19,35,39]. Our findings provide evidence of the long-term persistence of H5 subtypes in natural reservoirs with the changing dominance of subtypes. Continuous evolution of HPAI H5 viruses, via reassortment or antigenic shift, could pose a potential threat to human health if it acquires the ability to sustain human-to-human transmission. We argue that continuous surveillance in both domestic and wild birds is crucial to monitor the prevalence of different AIV subtypes and detect any cross transmission between wild and domestic birds. In Egypt, many factors may impact the prevalence and distribution of HPAI viruses, such as the density of birds and the weather. The cold weather might have favored the spread of both subtypes in the winter season, where a higher prevalence in the north of Egypt was found, compared to the warmer area in the south part of Egypt [40]. The national surveillance program that has been carried out in Egypt, depending on both active surveillance (collecting samples from farms, backyards, and live bird markets) and passive surveillance (reported cases by bird owners), has provided a robust data stream to uncover the full landscape of AIV dynamics. However, these surveillance efforts have been greatly influenced by the current COVID-19 pandemic regulations and restrictions.

## 5. Conclusions

In conclusion, we should remain vigilant and update our surveillance strategies to match current pandemic regulations. It is imperative that all poultry farmers should be aware of up-to-date biosafety and biosecurity measurements. Moreover, vaccination strategies in countries where AIV is endemic are important to reduce virus burden. In addition, regional and international cooperation through early warning systems is highly recommended to minimize the risk of virus transmission. Ultimately, an effective one health approach is important to deal with the emergence and control of avian influenza virus.

## Data Availability

Epidemiological data included in this study is available at https://empres-i.review.fao.org/#/, accessed on 27 April 2021 or available upon request from the author.

## Supplementary Materials

The following are available online at https://www.mdpi.com/1999-4915/13/8/1565/s1. Figure S1: Spatial hot spot analysis of global H5N1 outbreaks. Figure S2: Spatial hot spot analysis of global H5N8 outbreaks. Figure S3: Temporal dynamics and correlation between the number of global H5N1 and H5N8 outbreaks in 2015–2020 in domestic and wild birds. Figure S4: Correlation between the number of H5N1 and H5N8 positive samples in Egypt. Table S1. Positive cases of H5N1 and H5N8 in Egypt by sectors and years.
