# Supplementary material for: Temporal Dynamics of Influenza A(H5N1) Subtype before and after the Emergence of H5N8
Source: Viruses. 2021 Aug 7;13(8):1565. doi: 10.3390/v13081565 (PMC8412109; doi:10.3390/v13081565)
Supplement: Supplementary file 1 [file viruses-13-01565-s001.zip › viruses-1296875-supplementary.pdf]

Supplementary Materials: Temporal Dynamics of Influenza A(H5N1) Subtype Before and After the Emergence of H5N8

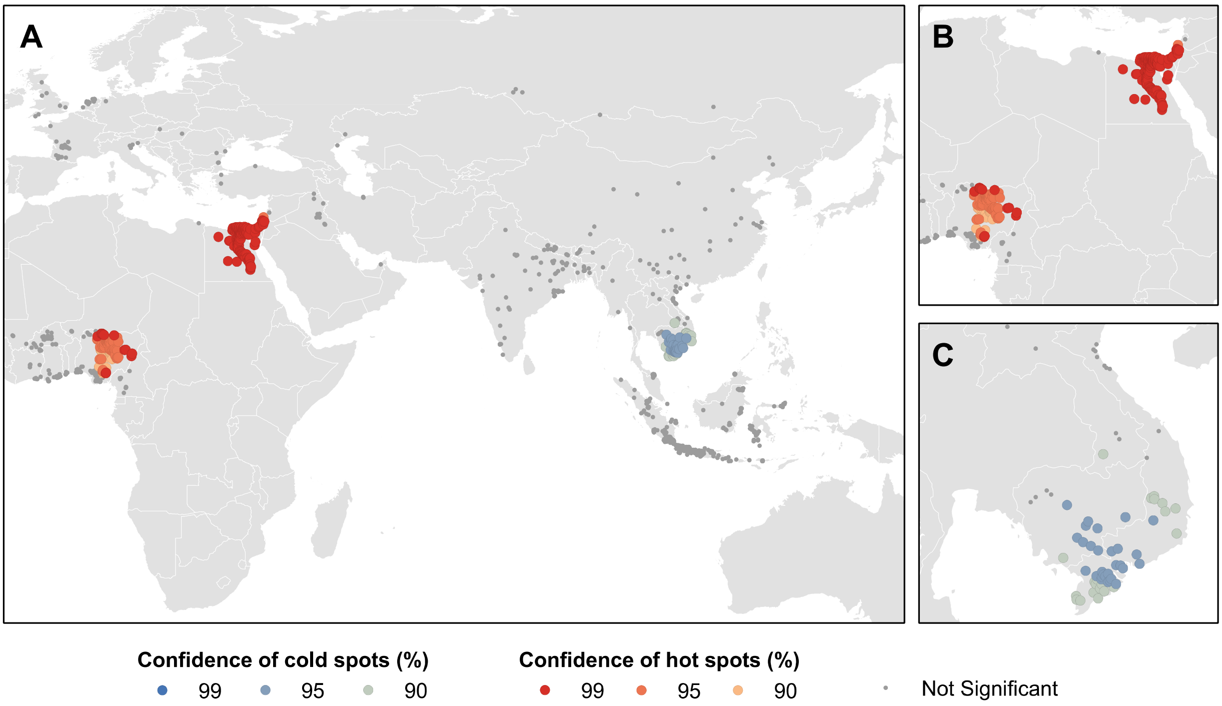

Supplementary Figure S1. Spatial hot spot analysis of global H5N1 outbreaks. Spatial clusters of (**A**) global H5N1 outbreaks were identified by using the Getis-Ord Gi* statistic. Spatial clusters were classified to differing levels of significance at the 99, 95 and 90 percent confidence level. Spatial clusters in (**B**) Africa and (**C**) Southeast Asia were highlighted.

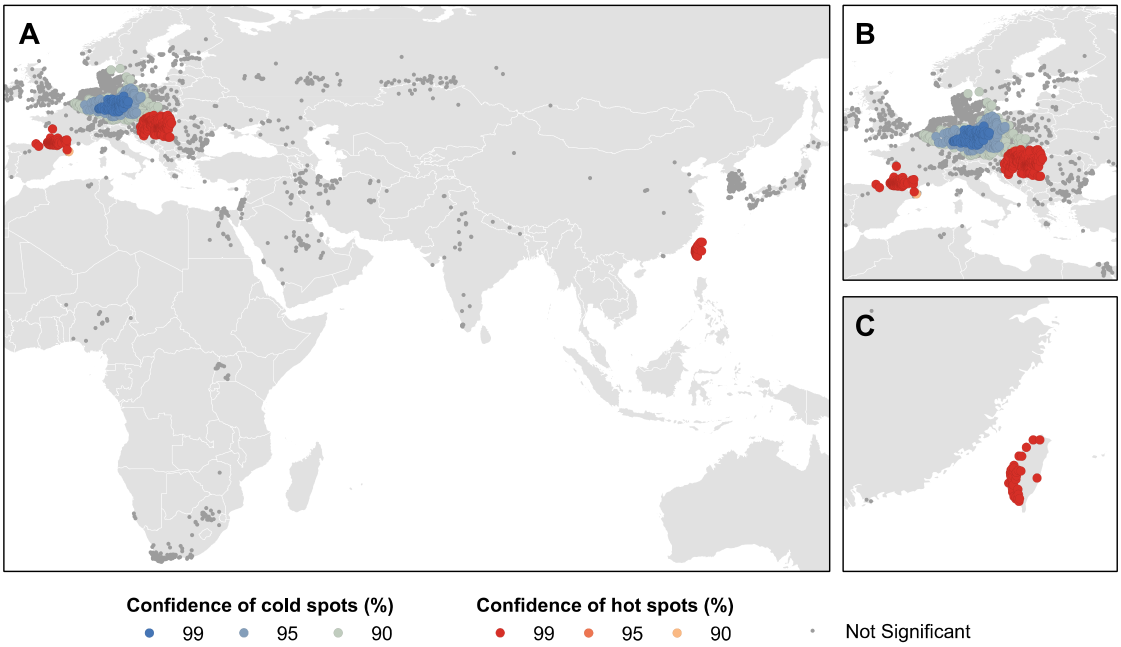

Supplementary Figure S2. Spatial hot spot analysis of global H5N8 outbreaks. Same with figure S1 but for (**A**) global H5N8 outbreaks. Spatial clusters in (**B**) Europe and (**C**) Taiwan, China were highlighted.

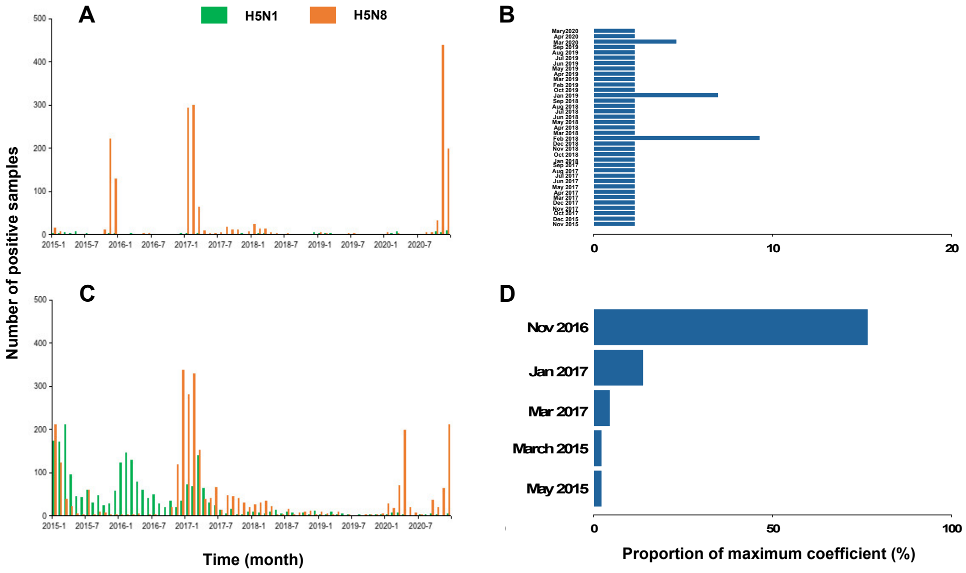

Supplementary Figure S3. Temporal dynamics and correlation between the number of global H5N1 and H5N8 outbreaks in 2015-2020 in domestic and wild birds. The (**A**) time series and (**B**) the distribution of the timing for the maximum correlation is provided for outbreaks in wild and (**C**,**D**) domestic birds.

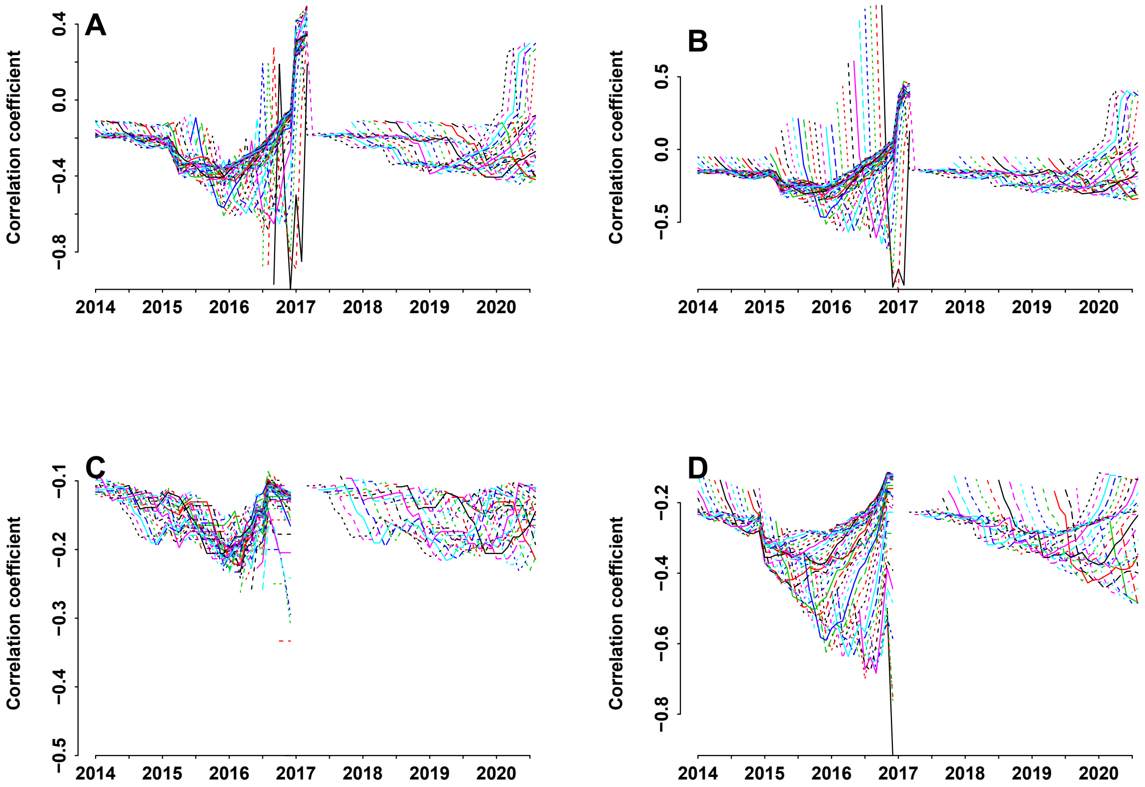

Supplementary Figure S4. **Correlation between the number of H5N1 and H5N8 positive samples in Egypt.** Correlation coefficients over time is estimated with varying window size. Each line shows the coefficients with a window size for (**A**) the overall positive samples and those in (**B**) backyards, (**C**) farms, (**D**) LBMs, respectively.

Supplementary Table S1. Positive cases of H5N1 and H5N8 in Egypt by sectors and years.

Subtype	total	By sector			By year						
		Backyards	Farms	LBMs	2014	2015	2016	2017	2018	2019	2020
H5N1	934	694	82	153	373	382	172	7	0	0	0
H5N8	322	121	67	132	0	0	2	83	93	85	59
